# Effect of Seminal Plasma from Good and Bad Freezer Stallions on Post-Thaw Acrosomal Integrity of SLC-Selected Spermatozoa

**DOI:** 10.3390/ani16152329

**Published:** 2026-07-30

**Authors:** Essraa M. Al-Essawe, Michaela Toni, Anders Johannisson, Jane M. Morrell

**Affiliations:** 1 Higher Institute for Infertility Diagnosis and Assisted Reproductive Technologies, Department of Physiology, Al-Nahrain University, Baghdad P.O. Box 70043, Iraq; essraa.m.al.essawe@st.nahrainuniv.edu.iq; 2 Department of Clinical Sciences, Swedish University of Agricultural Sciences (SLU), 750 07 Uppsala, Sweden; michaela.toni@live.se; 3 Department of Medical Sciences, Uppsala University, 751 85 Uppsala, Sweden; anders.johannisson@akademiska.se

**Keywords:** stallion semen, cryopreservation, SingleLayer Centrifugation, seminal plasma, acrosomal integrity, equine reproduction, acrosome reaction, flow cytometry

## Abstract

Freezability of stallion spermatozoa varies considerably between individuals. The aim was to investigate whether adding seminal plasma (SP) from good freezer or bad freezer stallions improved post-thaw sperm acrosomal integrity. Semen samples were available where SP-free spermatozoa from good freezer and bad freezer stallions prepared by SingleLayer Centrifugation (S) were treated with pooled SP from the same stallions prior to freezing. Control samples were frozen conventionally (C). Post-thaw acrosomal integrity was assessed by flow cytometry with/without stimulation by calcium ionophore. Higher proportions of live acrosome-intact spermatozoa (*p* = 0.04) and lower proportions of dead acrosome-intact spermatozoa (*p* = 0.48) occurred in non-stimulated S samples than in C. Acrosome integrity was not affected by either type of SP. However, in ionophore-stimulated samples, SP from good freezer stallions produced higher proportions of live acrosome-intact spermatozoa than C (*p* = 0.047), whereas SP from bad freezer stallions resulted in higher proportions of live acrosome-reacted spermatozoa than C (*p* = 0.04); the proportion of dead acrosome-intact spermatozoa was higher in C than in S (*p* = 0.05). Thus, S had a lower propensity to acrosome-react under stimulating conditions than C; the effect of adding SP depends on its origin. Further research is required to optimise cryopreservation protocols for stallion semen.

## Simple Summary

Freezing of stallion semen is challenging because of varying success between individuals. Sometimes, sperm have already acrosome-reacted during freezing and thawing, which means that they are no longer useful for artificial insemination. The variation between stallions could be due to the composition of their seminal plasma. Therefore, to investigate this effect, seminal plasma from good or bad freezer stallions was added back to seminal plasma-free sperm samples from the same stallions before freezing. Acrosome integrity was assessed after freezing and thawing. The results showed that seminal plasma does affect the readiness with which stallion sperm acrosome react. There were more sperm with intact acrosomes in seminal plasma-free sperm samples than controls frozen in their own seminal plasma. Adding seminal plasma from bad freezers increased the proportion of acrosome-reacted sperm, whereas adding seminal plasma from good freezers maintained acrosome integrity. These results may help to optimise protocols for stallion sperm freezing.

## 1. Introduction

Cryopreserved semen plays a central role in modern horse breeding and offers several advantages over fresh semen. Artificial insemination is widely used in animal reproduction [1], particularly for genetic improvement, and frozen-thawed stallion semen has become an essential component of contemporary breeding programmes [2,3]. A single ejaculate from a genetically superior stallion can be used to inseminate multiple mares, maximising the dissemination of desirable traits while reducing the risk of sexually transmitted diseases by eliminating direct contact [4]. In addition, frozen semen facilitates long-distance and international transport and preserves valuable genetics, even if a stallion later becomes unavailable [2].

Despite these advantages, artificial insemination with frozen semen in horses presents practical challenges. Fertility rates are often lower than with fresh semen, largely due to cryodamage and the high sensitivity of the timing of insemination of frozen semen relative to ovulation. Improvements in cryopreservation protocols are therefore essential. Successful semen freezing enhances reproductive efficiency, economic returns, and long-term conservation of genetic lines, and numerous strategies have been developed to reduce cryoinjury [5].

A major limitation to optimising protocols for cryopreservation is the considerable individual variation in sperm freezability among stallions [6,7,8]. Stallions are commonly classified as good freezers or bad freezers based on post-thaw sperm survival [6]. Only about 20–30% of stallions consistently produce high-quality frozen semen, whereas many produce semen of moderate quality or are unsuitable for cryopreservation despite being fertile under natural mating [4]. Breed-related differences have also been reported [7,8].

The non-cellular component of semen, seminal plasma, is secreted by the accessory sex glands; it provides metabolic support, facilitates sperm transport, and contributes to sperm maturation [9]. However, reducing seminal plasma is generally considered beneficial for semen preservation [6,10]. In stallion cryopreservation protocols, much of the seminal plasma is removed by centrifugation to concentrate spermatozoa, although this process can negatively affect viability, mitochondrial activity, and acrosomal integrity [11].

During freezing and thawing, spermatozoa experience cold shock and osmotic stress that disrupt membrane structure and function. These changes may trigger premature capacitation-like alterations and acrosome reactions, reducing fertilising ability [5].

One method to improve semen quality prior to freezing is colloid centrifugation, particularly SingleLayer Centrifugation. This technique selects a subpopulation of spermatozoa with superior functional characteristics while removing debris and damaged cells [12]. Compared with conventional centrifugation, SingleLayer Centrifugation was associated with improved chromatin integrity, mitochondrial function, and reduced oxidative stress after thawing [13], potentially increasing resistance to cryoinjury [14,15,16]. It has been recommended to enhance post-thaw progressive motility and to improve the suitability of semen from poor freezers [17].

The sperm acrosome, covering the anterior portion of the sperm head, contains enzymes essential for penetrating the oocyte’s zona pellucida during fertilisation [18,19]. Acrosomal integrity during cryopreservation is therefore critical for maintaining fertilising capacity [20]. One possible explanation for better cryosurvival in some stallions could lie in the ability of their seminal plasma to protect spermatozoa during cryopreservation. Vieira et al. [21] reported that ROS production was reduced when seminal plasma was added to epididymal spermatozoa; they attributed this effect to the composition of seminal plasma, which differed in protein concentration, antioxidant capacity, and polyunsaturated fatty acids between individuals. In a different study, addition of autologous seminal plasma improved sperm motility in some stallion semen samples but not in others [22].

We hypothesised that seminal plasma from good freezer stallions would better preserve acrosomal function and responsiveness than seminal plasma from bad freezer stallions. The objective of this study was to evaluate the effect of supplementing ejaculated SLC-selected stallion spermatozoa with 5% pooled seminal plasma from stallions of differing freezability (good vs. bad freezers) prior to cryopreservation on post-thaw acrosomal integrity. The concentration of 5% seminal plasma was based on previous studies demonstrating that low concentrations of seminal plasma can preserve beneficial components while minimising detrimental effects on sperm cryosurvival. Higher concentrations of seminal plasma have been associated with reduced sperm motility and membrane stability during cryopreservation. Therefore, the use of 5% seminal plasma represents a compromise that allows the reintroduction of physiologically relevant seminal plasma factors without excessive exposure to potentially harmful components [23]. 

We used sperm samples that had been processed for previous experiments, in which SP-free sperm samples were treated with SP from either good freezer or freezer stallions, since acrosome status was not evaluated previously [13]. The ability of these spermatozoa to bind to the zona pellucida was previously assessed in a heterologous zona binding assay [24]. 

## 2. Materials and Methods

**Figure 1:**
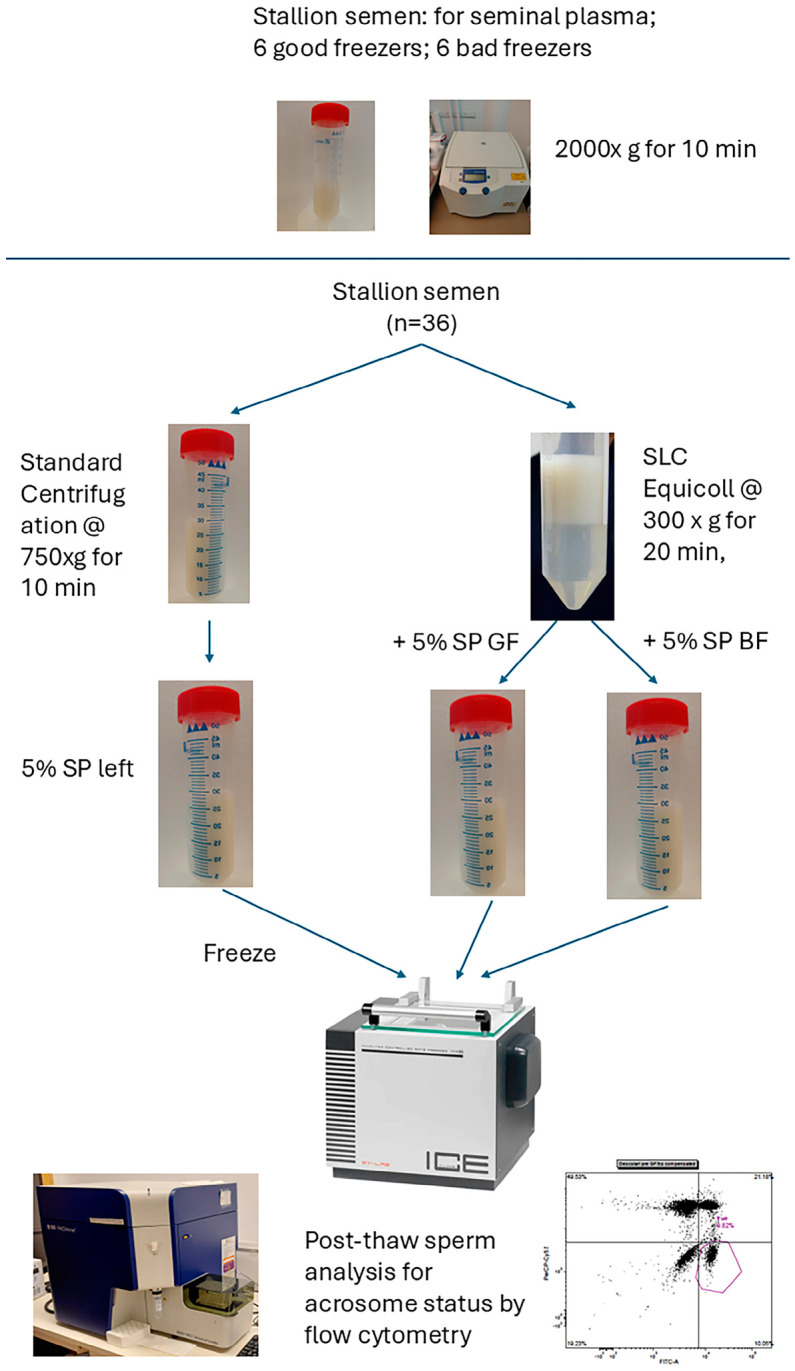
Pre-freezing treatment of stallion semen in a previous experiment [13]. Acrosome status was analysed post-thawing (n = 48). SP = seminal plasma; GF = good freezer stallions and BF = bad freezer stallions, based on the stud farm’s standard assessment of post-thaw motility.

The experimental design is shown in Figure 1.

The details of SP preparation, SLC and treatment of SLC samples with SP followed by freezing were described previously [13]. Similarly, the heterologous zona binding assay was detailed in full [24]. Brief details are presented here.

### 2.1. Semen Collection

Warmblood sporthorse stallions were kept under standard husbandry conditions at the Brandenburg State Stud, Neustadt Dosse, Germany, for the purposes of semen collection. This is a European Union-approved commercial AI centre meeting the requirements of the European Council directive 92/65/EEC. The stallions were fed hay, oats, concentrates (Cornmüsli, Marstall, Oberstaufen, Germany), and mineral supplements (Pferdemineral, Salvana, Ahlhorn, Germany). The amount of food was adjusted according to the body condition of each stallion. Water was available at all times. 

Semen was collected during January (approximately 4–6 weeks before the start of the breeding season) using a Hannover model artificial vagina (Minitube, Tiefenbach, Germany) warmed to 45–50 °C, after the stallion had mounted a phantom in the presence of a mare. The ejaculate was filtered through gauze into a sterilised measuring cylinder to remove the gel fraction.

### 2.2. Sperm Freezability Classification

Prior to the commencement of the study, a freezing test was carried out to classify the stallions as either good freezers or bad freezers, as described previously [13]. Sperm freezing was performed as previously reported in the same reference [13]. Briefly, extended semen (1:2) was centrifuged at 750× *g* for 10 min before the supernatant was removed using a vacuum pump. The sperm pellet was re-suspended in 1 mL of Gent freezing extender, comprising egg yolk, 5% glycerol, and antibiotics, according to the manufacturer’s website (Minitube, Tiefenbach, Germany). An equal volume of freezing extender was added once the sperm pellet was completely resuspended. Straws were filled and sealed using an automatic filling and sealing machine (MPP Uno; Minitüb, Tiefenbach, Germany). The semen was frozen in a computer-controlled freezer (Ice Cube 14S; Minitube, Tiefenbach, Germany). A gradual cooling phase (0.3 °C/min) from 20 °C to 5 °C was followed by a rapid cooling phase (10 °C/min), from 5 °C to −25 °C, and finally by a second rapid cooling phase (25 °C/min) to cool the sample from −25 °C to −140 °C. After one hour, the straws were removed from the Ice Cube and immediately immersed in liquid nitrogen (−196 °C); they were preserved in liquid nitrogen until required for analysis [13]. 

One week later one straw from each ejaculate was thawed at 37 °C for 30 s. Post-thaw total and progressive motility were evaluated using the AndroVision (Minitüb, Tiefenbach, Germany). 

### 2.3. Computer-Assisted Sperm Analysis

Sperm motility was analysed using a SpermVision instrument (version 3.5; Minitüb GmbH, Tiefenbach, Germany). An aliquot (5 μL) of each sperm sample was placed on a warm glass slide, coverslipped and transferred to the warm stage of the microscope at 38 °C. Sperm motility of >2000 spermatozoa (approximately 350 per field in eight fields) were analysed for total motility (TM; %), progressive motility (PM; %), average path velocity (VAP; μm/s), curvilinear velocity (VCL; μm/s), straight-line velocity (VSL; μm/s), straightness (STR; VSL/VAP), linearity (LIN; VSL/VCL), wobble (WOB; VAP/VCL), amplitude of lateral head deviation (ALH; μm) and beat cross frequency (BCF; Hz). Spermatozoa were considered be immotile if VAP < 20 and locally motile if VAP > 20 but < 30, STR < 0.5, VCL < 9. According to convention at the stud, the stallion was classified as a “good freezer” if the post-thaw total motility was ≥60% and the progressive motility was ≥40 and as a “bad freezer” if the post-thaw total motility was ≤50% and the progressive motility was ≤30 [13].

### 2.4. Preparation of Seminal Plasma

Twelve Warmblood stallions, 4 to 18 years old (mean ± SD 7.8 ± 4.3 years) of proven fertility were previously identified as good freezers or bad freezers according to sperm motility after freezing (see Section 2.2 and Section 2.3). Seminal plasma was prepared from 6 good freezer stallions and 6 bad freezer stallions by centrifuging the raw ejaculate at 2000× *g* for 10 min. The supernatant was aspirated and checked for the presence of sperm. If sperm were detected, the seminal plasma was centrifuged again until sperm-free. After filtering through a 0.2 μm filter, it was frozen and stored at −80 °C until required. The seminal plasma was pooled using equal volumes from good freezer stallions to create the good freezer seminal plasma pool and equal volumes of seminal plasma from bad freezer stallions to create the bad freezer seminal plasma pool [13].

### 2.5. Preparation of Sperm Samples

Ejaculates were collected from the same 12 stallions and were split; one portion of each ejaculate was prepared conventionally for freezing by extending with warm extender and centrifuging at 750× *g* for 10 min. The supernatant was removed, leaving the last 5 mL to resuspend the sperm pellet (control). The remaining ejaculate was adjusted to a sperm concentration of 100 × 10^6^/mL and was layered over 15 mL Equicoll (Minitube, Tiefenbach, Germany) [25]. After centrifuging for 20 min at 300× *g*, the supernatant was removed, and the sperm pellet was resuspended in EquiPlus (Minitube, Tiefenbach, Germany). One SLC aliquot from each ejaculate was treated with 5% seminal plasma from good freezer stallions (S-GF); another aliquot was treated with SP from bad freezers S-BF); the third aliquot was left without SP (S). All samples were frozen in 0.5 mL straws at a sperm concentration of 200 × 10^6^/mL [13].

### 2.6. Thawing and Capacitation Procedure

Frozen semen straws were thawed in a water bath at 37 °C for 30 s. An aliquot of semen (3 μL) was diluted in 300 μL of modified Whitten’s medium (MWM) under capacitating conditions, i.e., in the presence of 25 mM NaHCO_3_ and 7 mg/mL bSA [26,27]. This sample was then divided into two; one aliquot was stained immediately (non-stimulated sample), whereas calcium ionophore (1 μL, 5 mg/mL) was added to the other aliquot, which was incubated for 45 min at 37 °C before staining (stimulated samples).

### 2.7. Heterologous Zona Binding Assay

Salt-stored in vitro matured bovine oocytes were washed and equilibrated in PBS-poly vinyl alcohol (PVA) for 1 h at 38 °C in 5% CO_2_. The oocytes were transferred into four-well dishes containing 460 mL MWM with approximately 25 to 30 oocytes per well. The sperm suspension was added to give a final concentration of 5 × 10^6^ sperm cells/mL. The plates were incubated for 14 to 18 h at 38 °C in a 5% CO_2_ incubator, with 100% humidity.

After pipetting the sperm–oocyte complexes 3 to 5 times to remove loosely attached sperm cells, the complexes were rinsed three times using PBS-PVA and fixed in 2% (*v*:*v*) paraformaldehyde in PBS-PVA overnight at 4 °C, before transferring to Quench solution (67 mM Glycine, 103 mM NaCl and 21.2 mM NaH_2_PO_4_·H_2_O; 23) for storage. The oocytes were stored at 4 °C for up to 5 days before assessment [27,28].

### 2.8. Assessment of Sperm Binding

The oocyte complexes were washed three times with PBS-PVA and then moved to a 100 µL droplet PBS/PVA containing 2.5 mg/mL Hoechst 33342 (Sigma, St. Louis, MO, USA). After incubating for 20 to 30 min at room temperature (23 °C) in the dark, the complexes were mounted with anti-fade medium Vectashield (Vector Laboratories, Burlingame, CA, USA). The number of spermatozoa bound to each oocyte ZP was assessed using an epifluorescence microscope (LSM 510, Carl Zeiss, AB, Jena, Germany).

### 2.9. Acrosome Integrity

A FACSVerse flow cytometer (BDBiosciences, San José, CA, USA) was used to assess sperm acrosome integrity. Excitation of spermatozoa was achieved using a 488 nm blue laser. Green fluorescence from FITC-PNA was detected using a FL1 band-pass filter (527/32 nm), whereas red fluorescence from PI was detected using a FL3 band-pass filter (700/54 nm). Hoechst 33342 fluorescence was excited using a 405 nm violet laser and detected using the FL5 channel (528/45 nm band-pass filter). A total of 100,000 cells were analysed for each sample. Spermatozoa were classified into four populations based on acrosome integrity: living acrosome intact; living acrosome reacted; dead acrosome intact; and dead acrosome reacted [29]. The True live acrosome-reacted population was calculated as follows: live acrosome reacted under stimulated conditions minus live acrosome reacted under non-stimulated conditions [30].

### 2.10. Statistical Analyses 

Statistical analyses were performed using SAS^®^ Studio version 9.4. Data were first tested for normality using the Shapiro–Wilks test. Variables that did not follow a normal distribution were subjected to a logarithmic transformation to achieve approximate normality. Levene’s test was used to evaluate the homogeneity of variances. Means were compared using mixed-model ANOVA. The statistical model included: fixed effects of treatments (C, S, S-BF, S-GF) and freezability (good freezers vs. bad freezers). The treatment × freezability interaction and the random effect of individual stallion (n = 12) were also included. The effects of calcium ionophore stimulation and freezability were analysed using mixed-model ANOVA, including treatments (C, S, S-BF, S-GF), stimulation status (stimulated vs non-stimulated) and treatment × stimulation interaction. The random effect of stallion was included in the model. Tukey’s post hoc test was applied for multiple comparisons. A *p*-value ≤ 0.05 was considered statistically significant.

## 3. Results

Sperm quality in the original ejaculates was reported previously [31] and are summarised as follows (mean ± SD): semen volume 20.8 ± 2.2 mL, sperm concentration 348 × 10^6^ ± 23 × 10^6^; normal morphology 68.4 ± 3.0%, total motility 77.3 ± 4.8%, progressive motility 55.1 ± 5.%.

In non-stimulated samples from bad freezer stallions, (Table 1), S samples had a higher proportion of LAI spermatozoa than C (*p* = 0.04) and lower DAI (*p* = 0.048). For good freezer stallions in non-stimulated samples, DAI was higher in C than in S (0.001). 

For bad freezer stallions in stimulated samples (Table 2) the S samples showed significantly higher proportions of live acrosome-intact spermatozoa than the controls (*p* = 0.047); furthermore, S-BF had a higher proportion of live acrosome-reacted spermatozoa than the controls (*p* = 0.04). The S samples had lower dead acrosome-reacted spermatozoa than the controls (*p* = 0.05). No significant differences between treatments were observed for dead acrosome-reacted spermatozoa.

When the results from bad freezers and good freezers were combined (Table 3), the proportion of live acrosome-reacted spermatozoa from non-stimulated samples were higher for S, S-BF and S-GF than for the controls (*p* < 0.05). In stimulated samples, S, S-BF and S-GF also had a higher proportion of live acrosome-reacted spermatozoa than the controls (*p* < 0.05). As anticipated, the proportion of live acrosome-reacted spermatozoa for all groups was higher in stimulated samples than in non-stimulated samples (*p* = 0.001).

The acrosome integrity results for six of the stallions from the present study are shown in Table 4 (three good freezers and three bad freezers), together with the results of the same samples in the heterologous zona binding assay reported previously [24].

**Figure 2:**
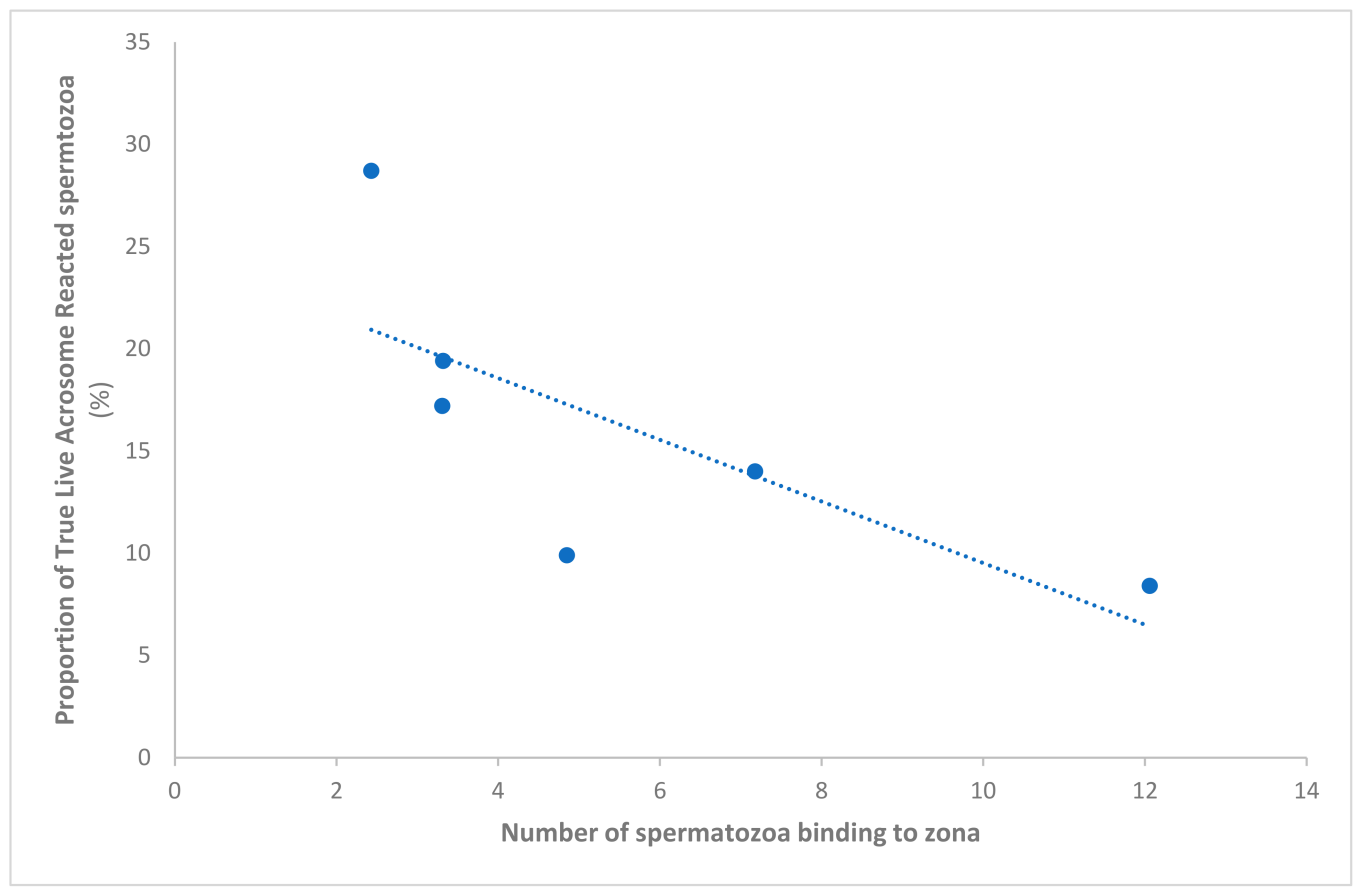
Relationship between True live acrosome-reacted stallion spermatozoa under stimulation of calcium ionophore and the number of spermatozoa binding to the zona pellucida of bovine oocytes (n = 24).

There was a negative correlation (r = −0.736; *p* < 0.05) between True live acrosome-reacted spermatozoa for S-GF samples and binding to the zona pellucida of bovine oocytes (Figure 2). No significant correlations were found between acrosome status and zona binding for the other treatment groups.

## 4. Discussion

The aim of this study was to investigate whether the addition of 5% seminal plasma from stallions classified as good freezers (or bad freezers) to seminal plasma-free stallion spermatozoa prior to cryopreservation could enhance post-thaw acrosomal integrity, potentially improving fertility outcomes when using frozen semen. The proportion of live acrosome-intact spermatozoa in control samples was similar to another study on stallion semen cryopreservation, where approximately 25% of the thawed spermatozoa had intact acrosomes [32]. The results demonstrated that SingleLayer Centrifugation significantly improved post-thaw acrosomal integrity. Specifically, for bad freezer stallions, the proportion of live spermatozoa with intact acrosomes) was increased in SingleLayer Centrifugation samples compared to controls under non-stimulating conditions, and the proportion of dead spermatozoa with intact acrosomes under stimulation with calcium ionophore was decreased compared with conventional centrifugation (the controls). These findings are consistent with previous studies reporting that SingleLayer Centrifugation improves post-thaw sperm quality by selecting spermatozoa with intact chromatin, better mitochondrial function, and improved motility [12,13,16].

Interestingly, the addition of SP from either good freezer or bad freezer stallions did not improve acrosomal integrity under non-stimulated conditions. However, the addition of 5% seminal plasma SP from bad freezer stallions resulted in a higher proportion of live acrosome spermatozoa following stimulation with calcium ionophore compared with the control group. This observation requires careful interpretation, as spermatozoa must undergo the acrosome reaction at the appropriate time and location for successful fertilisation. Under physiological conditions, spermatozoa should remain acrosome-intact prior to reaching the oviduct but retain the capacity to undergo the acrosome reaction in response to appropriate stimuli. Therefore, an increased proportion of acrosome-reacted spermatozoa after artificial stimulation in the bad freezer group in the present study may indicate premature acrosome loss. In contrast, Tello-Mora et al. [30] reported a positive correlation between blastocyst development rate and the proportion of acrosome-reacted sperm following stimulation of human spermatozoa. From our results, it is possible that seminal plasma from good freezer stallions contains factors that support sperm survival and prevent capacitation until the appropriate conditions are met. Further studies are needed to determine whether different SP concentrations or sources could produce more pronounced effects under stimulated conditions [30] or whether it is better to remove all seminal plasma with SingleLayer Centrifugation.

Another important aspect of sperm function is the ability to bind to the zona pellucida, which is essential for successful fertilisation. Our previous study showed that fewer sperm bound to the zona pellucida after the addition of seminal plasma SP from good freezer stallions [24]. One possible explanation is that if seminal plasma from good freezer stallions protects sperm from premature capacitation, it may also reduce immediate zona binding capacity. This delay might be beneficial in vivo, where there is only a narrow window around ovulation to achieve fertilisation when thawed sperm samples are inseminated.

The reduction in zona binding observed in previous studies after seminal plasma addition suggests that seminal plasma from good freezer stallions may contain decapacitation factors that delay capacitation. Such a delay may prolong sperm longevity in the female reproductive tract but could temporarily reduce fertilising capacity in vitro. Similar findings have been reported for seminal plasma components such as cysteine-rich secretory protein 1D (CRISP-1D), which has been shown to exert decapacitation effects and prevent premature capacitation [33,34]. In contrast, SP from BF stallions did not show the same inhibitory effect, possibly due to differences in seminal plasma composition [35,36].

Given the dual role of seminal plasma in sperm survival and function, its use prior to cryopreservation requires careful optimisation to balance its protective effects with potential reductions in fertilisation capacity. Customising seminal plasma supplementation based on individual stallion characteristics has previously been recommended to improve cryopreservation outcomes [37,38]. One of the problems with using thawed stallion spermatozoa for insemination is the need to deposit the sperm sample as close to ovulation as possible. Adding seminal plasma from good freezer stallions might provide greater flexibility by extending the time needed for sperm capacitation, thus increasing the window around ovulation when insemination could be performed. 

One limitation of the present study is that semen was collected in January during the non-breeding season, approximately 6 weeks prior to the commencement of the breeding season. Although stallions continue to produce spermatozoa throughout the year, seasonal variations can influence semen quality and reproductive parameters. In a study in Switzerland, autumn was identified as being the best time to freeze stallion semen [39]. However, only small differences between the breeding and non-breeding seasons were observed in stallions in Germany by [31], whereas Suliman et al. [40] found differences but only between fertile and sub-fertile stallions, rather than between the breeding and non-breeding seasons. Reactive oxygen species production by spermatozoa were reported to differ between the start and conclusion of the breeding season in Sweden [41]. In contrast, marked differences in sperm quality were seen between the breeding and non-breeding seasons in Spain [42], although the authors did also note considerable individual variation. These apparent contrasts may reflect differences in climate due to geographical location, individual variation and the type of analysis performed. 

Reduced testicular activity and lower sperm output may occur during the non-breeding season in some stallions, which could potentially influence sperm cryosurvival. However, the stallions were managed under similar environmental and management conditions, minimising variability within the study. It should be noted that it is customary to freeze semen during the non-breeding season when there is no demand for fresh semen for artificial insemination [43,44]. In fact, Mislei et al. recommend freezing during the non-breeding season since chromatin damage is lower than in the non-breeding season [45]. A study on sperm freezability in Chilean heavy horses indicated that sperm cryosurvival was better in ejaculates collected during the non-breeding season [46]. Therefore, it was important to conduct this study under the same conditions that would prevail during standard freezing. A previous study on seminal plasma proteins, taken at the same time and from the same animals as the samples in the present study, revealed only minor differences in seminal plasma protein composition between the breeding and non-breeding seasons [31]. The authors of the study on Chilean heavy horses suggested that there could be cryoprotective factors in the seminal plasma during the non-breeding season [46]. In contrast, another study identified differences in kallikreins in seminal plasma of Mangalarga Marchador stallions in Brazil [47], where the authors suggested that kallikrein might negatively influence sperm quality in the non-breeding season. Nevertheless, future studies conducted during the breeding season may provide additional insight into the potential influence of seasonal factors on sperm freezability and seminal plasma effects.

Another limitation of this study is the use of pooled seminal plasma, which may obscure the effects of individual seminal plasma samples. Previous research has demonstrated that the biological activity of seminal plasma is strongly dependent on its source [13,48]. Seminal plasma composition varies considerably between stallions [21,49] and contains proteins such as CRISP-3 and fibronectin, which are known to influence sperm motility and freezability [48,50]. Furthermore, Vieira et al. [21] suggested that characterising seminal plasma composition could help identify stallions with superior cryopreservation potential, thereby improving reproductive efficiency in breeding programmes [21].

Excessive exposure to SP before freezing has also been associated with detrimental effects on sperm quality [20,23,51]. Some seminal plasma components, such as lactoferrin, may reduce inflammatory responses and protect spermatozoa from oxidative stress during cryopreservation [52]. Therefore, the overall impact of seminal plasma likely depends on the balance between beneficial and detrimental factors. The addition of 5% seminal plasma in the study reported here was based on the presumed seminal plasma content of samples prepared by conventional centrifugation. It would be interesting to repeat the study with other proportions of seminal plasma.

Finally, it should be noted that the stimulation of spermatozoa using calcium ionophore does not fully replicate physiological conditions within the female reproductive tract [53]. While responsiveness to calcium ionophore stimulation may reflect functional competence, premature acrosome reaction could negatively affect fertilisation in vivo. The limitations of calcium ionophore assays in mimicking physiological conditions have also been acknowledged [54].

The results from the present study suggest that the interaction between seminal plasma and spermatozoa from good and bad freezer stallions is complex. If the status of the stallion is unknown, i.e., whether it is a good or bad freezer, it might be best to remove all the seminal plasma with SingleLayer Centrifugation prior to freezing. If the stallion is known to be a bad freezer, there might be some benefit in adding up to 5% seminal plasma from a good freezer stallion after removal of the stallion’s own seminal plasma to increase the availability of non-acrosome-reacted spermatozoa post-ovulation. However, if the thawed spermatozoa are to be used for in vitro fertilisation, adding 5% seminal plasma from a bad freezer stallion prior to freezing might speed up the acrosome reaction after thawing. 

Future studies should investigate the effects of different seminal plasma concentrations and evaluate the impact of alternative semen extenders on acrosomal integrity. Identifying specific SP components responsible for the observed effects such as CRISP-3, fibronectin, and lactoferrin may contribute to the development of improved cryopreservation strategies and enhance post-thaw sperm function.

## 5. Conclusions

This study demonstrates that SingleLayer Centrifugation prior to freezing significantly improves post-thaw acrosomal integrity in stallion spermatozoa compared with conventional centrifugation. Although the addition of seminal plasma did not significantly enhance acrosomal integrity under non-stimulated conditions, seminal plasma from bad freezer stallions increased the proportion of acrosome-reacted spermatozoa following stimulation, whereas seminal plasma from good freezer stallions protected against acrosome reaction. It may be possible to exploit these findings to extend the window around ovulation when insemination with thawed semen is needed. These findings highlight the complex role of seminal plasma in sperm cryopreservation and emphasise the need for careful optimisation of seminal plasma supplementation. Future research should focus on identifying specific seminal plasma components responsible for these effects to develop more targeted cryopreservation protocols and improve reproductive outcomes in equine breeding programmes. 

## Figures and Tables

**Table 1:** Post-thaw acrosomal integrity of non-stimulated stallion spermatozoa after cryopreservation using single-layer centrifugation (SLC) and seminal plasma from good and bad freezer stallions (means ± sem; n = 12 stallions).

Groups	Bad Freezer Stallion				Good Freezer Stallion			
	LAI%	LAR%	DAI%	DAR%	LAI%	LAR%	DAI%	DAR%
C	16.13 **^A^** ± 4.65	0.33 ± 0.18	53.53 **^B^** ± 4.75	30.52 ± 4.08	19.16 ± 4.65	0.35 ± 0.18	56.57 **^C^** ± 4.75	23.92 ± 4.08
S	27.59 **^A^** ± 4.65	0.49 ± 0.18	41.76 **^B^** ± 4.75	30.16 ± 4.08	29.52 ± 4.65	0.51 ± 0.18	42.57 **^C^** ± 4.75	27.40 ± 4.08
S-BF	22.23 ± 4.65	0.47 ± 0.18	48.69 ± 4.75	28.59 ± 4.08	26.52 ± 4.65	0.62 ± 0.18	48.15 ± 4.75	24.71 ± 4.08
S-GF	24.79 ± 4.65	0.41 ± 0.18	45.42 ± 4.75	29.39 ± 4.08	27.79 ± 4.65	0.56 ± 0.18	47.43 ± 4.75	24.21 ± 4.08

Note: LAI: living acrosome intact, LAR: living acrosome reacted, DAI: dead acrosome intact, DAR dead acrosome-reacted (DAR) spermatozoa. Control (C) = standard centrifugation, S = SingleLayer Centrifugation (SLC) without seminal plasma, S-GF = SLC with 5% seminal plasma from good freezer stallions, S-BF = SLC with 5% seminal plasma from bad freezer stallions. Significant differences between treatments are indicated by different superscript letters (*p* ≤ 0.05). Same capital letters within columns indicate significant differences as follows: A *p* = 0.024; B *p* = 0.048; C *p* = 0.01.

**Table 2:** Post-thaw acrosomal integrity of stimulated stallion spermatozoa after cryopreservation and calcium ionophore stimulation (means ± sem; n = 12 stallions).

Groups	Bad Freezer Stallion				Good Freezer Stallion			
	LAI%	LAR%	DAI%	DAR%	LAI%	LAR%	DAI%	DAR%
Stim C	12.41 **^A^** ± 3.32	3.15 **^B^** ± 1.05	50.29 **^C^** ± 3.34	34.15 ± 3.11	18.05 ± 3.32	4.24 ± 1.05	55.10 ± 3.34	22.61 ± 3.11
Stim S	20.07 ± 3.32	4.70 ± 1.05	40.96 **^C^** ± 3.34	34.27 ± 3.11	23.37 ± 3.32	4.54 ± 1.05	46.08 ± 3.34	26.05 ± 3.11
Stim S-BF	17.58 ± 1.66	6.65 **^B^** ± 1.05	44.27 ± 3.34	31.50 ± 3.11	20.74 ± 3.32	5.22 ± 1.05	47.55 ± 3.34	26.50 ± 3.11
Stim S-GF	19.91 **^A^** ± 3.32	5.33 ± 1.05	43.07 ± 3.34	31.69 ± 3.11	21.88 ± 3.32	4.48 ± 1.05	47.65 ± 3.34	25.99 ± 3.11

Note: Stim = stimulated with calcium ionophore; LAI: living acrosome intact, LAR: living acrosome reacted, DAI: dead acrosome intact, DAR: dead acrosome-reacted (DAR) spermatozoa. Control (C) = standard centrifugation, S = SingleLayer Centrifugation (SLC) without seminal plasma, S-GF = SLC with 5% seminal plasma from good freezer stallions, S-BF = SLC with 5% seminal plasma from bad freezer stallions. Significant differences between treatments are indicated by different superscript letters (*p* ≤ 0.05). Same superscript letters within columns indicate significant differences as follows: A *p* = 0.047; B *p* = 0.04; C *p* = 0.05.

**Table 3:** Comparative analysis of acrosomal integrity in non-stimulated and stimulated stallion spermatozoa post-thaw (means ± sem; n = 12 stallions).

Groups	Non-Stimulated Samples				Stimulated Samples			
	LAI%	LAR%	DAI%	DAR%	LAI%	LAR%	DAI%	DAR%
C	17.76 ± 2.77	0.34 **^AD^** ± 0.52	55.05 ± 2.82	27.22 ± 2.64	15.23 ± 2.77	3.69 **^BD^** ± 0.52	52.69 ± 2.82	28.38 ± 2.64
S	28.55 ± 2.77	0.5 **^AD^** ± 0.52	42.16 ± 2.82	28.78 ± 2.64	21.70 ± 2.77	4.62 **^BD^** ± 0.52	43.52 ± 2.82	30.16 ± 2.64
S-BF	24.37 ± 2.77	0.55 **^AD^** ± 0.52	48.42 ± 2.82	26.65 ± 2.64	19.16 ± 2.77	5.93 **^BD^** ± 0.52	45.91 ± 2.82	29.00 ± 2.64
S-GF	26.29 ± 2.77	0.48 **^AD^** ± 0.52	46.43 ± 2.82	26.79 ± 2.64	20.89 ± 2.77	4.91 **^BD^** ± 0.52	45.36 ± 2.82	28.84 ± 2.64

Note: LAI: living acrosome intact, LAR: living acrosome reacted, DAI: dead acrosome intact, DAR: dead acrosome-reacted (DAR) spermatozoa. Control (C) = standard centrifugation, S = single-layer centrifugation (SLC) without seminal plasma, S-GF = SLC with 5% seminal plasma from good freezer stallions, S-BF = SLC with 5% seminal plasma from bad freezer stallions. Significant differences between treatments are indicated by the same superscript letters in the column (A, B: *p* ≤ 0.05). Same superscript letters across rows indicate significant differences as follows: D: *p* = 0.0001.

**Table 4:** Acrosome integrity in sperm samples from three good freezer and three bad freezer stallions from the present study, as well as heterologous binding assay results from a previous study (21). The sperm samples were prepared either by conventional centrifugation (C) or by single-layer centrifugation (S); S samples were left untreated or treated with seminal plasma from good freezer stallions or bad freezer stallions prior to freezing (n = 24).

Stallion ID	Treatment Group	LAR Stim (%)	LAR Non-Stim (%)	True AR (%)	Bound Sperm (n)
1	BF	3.53	0.27	3.26	4.8
2	BF	10.67	0.23	10.44	20
3	BF	5.39	1.99	3.4	36.7
4	BF	3.83	0.41	3.42	46
5	BF	3.41	0.23	3.18	34.4
6	BF	4.8	0.29	4.51	8.9
1	C	4.65	0.11	4.54	18.9
2	C	3.27	0.17	3.1	21.5
3	C	5.34	0.93	4.41	27.5
4	C	2.55	0.28	2.27	23
5	C	2.12	0.17	1.95	16
6	C	5.71	0.25	5.46	19
1	GF	5.11	0.26	4.85	9.9
2	GF	12.57	0.51	12.06	8.4
3	GF	5.1	1.78	3.32	19.4
4	GF	3.48	0.17	3.31	17.2
5	GF	2.81	0.38	2.43	28.7
6	GF	7.5	0.32	7.18	14
1	S	3.78	0.47	3.31	34.2
2	S	12.48	0.8	11.68	20.9
3	S	3.87	0.99	2.88	30.8
4	S	3.78	0.3	3.48	27.6
5	S	5.8	0.47	5.33	5.2
6	S	4.23	0.29	3.94	7

Note: LAR: living acrosome reacted, DAI: dead acrosome intact, DAR: dead acrosome-reacted (DAR) spermatozoa. Control = standard centrifugation, S = SingleLayer Centrifugation (SLC) without seminal plasma, S-GF = SLC with 5% seminal plasma from good freezer stallions, S-BF = SLC with 5% seminal plasma from bad freezer stallions. True AR = LAR stimulated minus LAR unstimulated; bound sperm = spermatozoa binding to the zona pellucida in a heterologous zona binding assay [24]. Each row represents one sample.

## Data Availability

The original contributions presented in this study are included in the article. Further inquiries can be directed to the corresponding author.
